# Higher plasma levels of lysophosphatidylcholine 18:0 are related to a lower risk of common cancers in a prospective metabolomics study

**DOI:** 10.1186/s12916-016-0552-3

**Published:** 2016-01-28

**Authors:** Tilman Kühn, Anna Floegel, Disorn Sookthai, Theron Johnson, Ulrike Rolle-Kampczyk, Wolfgang Otto, Martin von Bergen, Heiner Boeing, Rudolf Kaaks

**Affiliations:** Division of Cancer Epidemiology, German Cancer Research Center (DKFZ), Im Neuenheimer Feld 581, D-69120 Heidelberg, Germany; Department of Epidemiology, German Institute of Human Nutrition Potsdam-Rehbruecke, Arthur-Scheunert-Allee 114, D-14558 Nuthetal, Germany; Department of Metabolomics, Helmholtz Centre for Environmental Research (UFZ), Permoserstraße 15, D-04318 Leipzig, Germany; Department of Proteomics, Helmholtz Centre for Environmental Research (UFZ), Permoserstraße 15, D-04318 Leipzig, Germany; University of Aalborg, Fredrik Bajers Vej 7H, 9220 Aalborg East, Denmark

**Keywords:** Metabolomics, Epidemiology, Breast cancer, Prostate cancer, Colorectal cancer

## Abstract

**Background:**

First metabolomics studies have indicated that metabolic fingerprints from accessible tissues might be useful to better understand the etiological links between metabolism and cancer. However, there is still a lack of prospective metabolomics studies on pre-diagnostic metabolic alterations and cancer risk.

**Methods:**

Associations between pre-diagnostic levels of 120 circulating metabolites (acylcarnitines, amino acids, biogenic amines, phosphatidylcholines, sphingolipids, and hexoses) and the risks of breast, prostate, and colorectal cancer were evaluated by Cox regression analyses using data of a prospective case-cohort study including 835 incident cancer cases.

**Results:**

The median follow-up duration was 8.3 years among non-cases and 6.5 years among incident cases of cancer. Higher levels of lysophosphatidylcholines (lysoPCs), and especially lysoPC a C18:0, were consistently related to lower risks of breast, prostate, and colorectal cancer, independent of background factors. In contrast, higher levels of phosphatidylcholine PC ae C30:0 were associated with increased cancer risk. There was no heterogeneity in the observed associations by lag time between blood draw and cancer diagnosis.

**Conclusion:**

Changes in blood lipid composition precede the diagnosis of common malignancies by several years. Considering the consistency of the present results across three cancer types the observed alterations point to a global metabolic shift in phosphatidylcholine metabolism that may drive tumorigenesis.

**Electronic supplementary material:**

The online version of this article (doi:10.1186/s12916-016-0552-3) contains supplementary material, which is available to authorized users.

## Background

The simultaneous measurement of a large variety of small molecules in accessible tissues by metabolomics techniques may facilitate the identification of metabolic fingerprints of different cancers [1]. First prospective metabolomics studies have shown that elevations in circulating branched-chained amino acids 2–5 years prior to diagnosis are related to increased risk of pancreatic adenocarcinoma [2], that significant differences in serum levels of phosphatidylcholines and fatty acids between later prostate cancer cases and controls can be detected [3], and that pre-diagnostic serum concentrations of glycochenodeoxycholate are associated with colon cancer risk [4]. A complex pattern of pre-diagnostic metabolites representing several pathways has been identified in another prospective study on hepatocellular carcinoma [5]. For other cancers, differences in plasma metabolite concentrations between cases and controls have also been demonstrated, but prospective data are missing [1].

Here, we report results of the largest prospective metabolomics study so far on cancers of the breast, colorectum, and prostate. Our goal was the identification of metabolites that may give insight into metabolic alterations preceding the manifestation of cancer. In a case-cohort subset of the prospective population-based EPIC-Heidelberg study, we analyzed pre-diagnostic levels of 120 plasma metabolites by liquid chromatography-tandem mass spectrometry (LC-MS/MS) and flow injection analysis-tandem mass spectrometry (FIA-MS/MS), and evaluated associations between these metabolites and cancer risk over time.

## Methods

### Study population

EPIC-Heidelberg was established as part of the European Prospective Investigation into Cancer and Nutrition (EPIC) between 1994 and 1998 [6]. Overall, 25,540 adults (53.3 % females) aged 35–65 years from the local general population entered the study [7]. The vast majority of participants are white. Detailed information on habitual diet, smoking, alcohol consumption, physical activity, and socio-economic status was obtained by questionnaires and during interviews. In addition, anthropometric measurements were taken by trained personnel. Blood was drawn, processed, and aliquoted into 0.5 ml straws of serum (8 straws), plasma (12 straws), buffy coat (4 straws), and erythrocytes (4 straws), which were stored in liquid nitrogen at −196 °C. Plasma straws used for the present study were retrieved for the first time, i.e. they had not undergone freeze-and-thaw cycles.

Since the baseline, participants are followed-up by active and passive procedures [8]. Self-reported and registry-derived incident cases of cancer were validated by study physicians who accessed diagnostic records provided by treating physicians and hospitals. A case-cohort design [9] was chosen for the present metabolomics study and metabolites were measured in pre-diagnostic blood samples of cancer cases and a random subcohort [10]. Individuals in the case-cohort selection were free of diabetes at baseline, as the subcohort was initially drawn for the EPIC-InterAct study on incident diabetes [10]. All incident primary cases of breast (ICD-10 C50), prostate (ICD-10 C61), and colorectal cancer (ICD-10 C18–20) as well as cases secondary to non-melanoma skin cancer that had occurred before 31 December 2006 were selected. After exclusion of participants with missing covariate information (n = 4) and prevalent cancer cases (n = 65), the subcohort comprised 774 individuals. The final numbers of incident cases of breast, prostate, and colorectal cancer were 362, 310, and 163, respectively, after exclusions due to missing covariate information (breast cancer: n = 3; colorectal cancer: n = 2).

### Ethics

The present study was approved by the Ethics Committee of the Medical Faculty of the University of Heidelberg (Heidelberg, Germany). The study participants provided written consent for the use of their blood samples and data.

### Laboratory analyses

Metabolites, i.e. 40 acylcarnitines, 21 amino acids, 14 biogenic amines and creatinine, 76 phosphatidylcholines (PCs), 14 lysophosphatidylcholines (lysoPCs), 15 sphingomyelins, and overall hexose were quantified using the MetaDisIDQ™ Kit (Biocrates, Innsbruck, Austria). Sample preparation and measurements were carried out according to the suppliers’ protocol and have been described in detail elsewhere [11]. In short, 10 μl of standards, quality controls, and plasma samples, which had been thawed for 30 minutes at −4 °C, were pipetted onto filter paper within each well of the 96-well plate that had been commercially prepared. Nitrogen gas was used to dry the samples followed by application of phenylisothiocyanate (PITC) and extraction of metabolites from the filter paper. The eluate was then dried under flowing nitrogen gas and the wells sealed. Kit plates were shipped to the partner laboratory at the Department of Proteomics, Helmholtz Centre for Environmental Research (Leipzig, Germany), where liquid FIA-MS/MS (amino acids and biogenic amines) and LC-MS/MS (lipids and hexoses) analyses were carried out on each kit. Samples were blinded to the laboratory team. Metabolites were detected using an Agilent 1100 HPLC (Agilent Technologies, Böblingen, Germany) coupled to an API 5000 triple quadrupole mass spectrometer (AB Sciex, Darmstadt, Germany).

The MetaDisIDQ™ software (Biocrates) was used to randomize samples of the study population so that samples of cases and non-cases were stratified within and across batches. Each analytical batch contained three quality control (QC) samples of lyophilized human EDTA-plasma with standardized low, medium (spiked), and high (spiked) concentrations of internal standards to monitor the validity of measurements. Two blinded pooled QC plasma samples were included in duplicate to independently verify the consistency of measurements within and across batches. In addition to a commercially available pooled QC sample (NIST SRM 1950), we used a pooled sample from our own population. One blank without internal standard was included on each batch to calculate background levels and to check the system for contamination. Three additional zero samples with internal standards, but no analytes were integrated to calculate limits of detection.

### Data pre-processing and statistical analyses

Metabolites with more than 25 % missing values (n = 2), within batch coefficients of variation greater than 25 % (n = 12), or more than 25 % of values below the limit of detection (n = 47) were excluded so that 120 out of 181 metabolites were used for statistical evaluation (Additional file 1). All metabolite values were then log2-tranformed and normalized by metabolite-wise batch-standardization [12]. Multivariable outliers were identified by robust principal components analyses and excluded [13]. Spearman’s rho adjusted for age and sex was calculated to detect correlations between metabolites. Associations between metabolite concentrations and epidemiological covariates were assessed by calculating Spearman’s correlations (continuous covariates) and geometric mean values across categories (categorical covariates). Cox proportional hazards regression analyses were carried out to evaluate the relationships between circulating metabolites and cancer risks. Metabolite concentrations were divided into quartiles and linear trends were tested for using log2-transformed metabolite values on the continuous scale. The so-called Prentice method was used to account for the case-cohort design of the study [14]. Age was used as the underlying timescale, with age at recruitment as age at entry and age at the end of follow-up, death, or cancer diagnosis, whichever came first, as age at exit. The proportional hazards assumption was tested based on Schoenfeld residuals [15]. With respect to the vast majority of metabolites, and particularly those which showed significant associations with cancer risk, no strong violations of the proportional hazards assumption were noted. Adjustment factors for multivariable Cox regression models were selected based on literature review. The Bonferroni method was used to adjust for multiple testing, i.e. *P* values of single tests were divided by the total number of tests (n = 120). All statistical tests were two-sided and *P* values below 0.05 were considered statistically significant. All analyses were performed using SAS 9.3 (SAS Institute, Cary, NC, USA).

## Results

Characteristics of the study population are shown in Table 1. The case-cohort sample comprised a random subcohort (n = 774) and cases of breast (n = 362), prostate (n = 310), and colorectal cancer (n = 163). Median follow-up durations were 8.34 years in the subcohort and 6.36 years (breast cancer), 6.57 years (colorectal cancer), 6.83 years (prostate cancer), and 6.48 (overall cancer) among incident cases.

**Table 1:** Characteristics of the study population (EPIC-Heidelberg, case-cohort sample)

	Incident cancer cases			Subcohort	
	Colorectum	Breast	Prostate	Women	Men
N	163	362	310	426	348
Age at recruitment (years)	55.8 ± 6.4	51.4 ± 7.8	57.9 ± 5.2	49.1 ± 8.5	52.3 ± 7.1
Women (%)	37.4	100			
Menopausal status (%)					
Premenopausal		31.5		48.6	
Postmenopausal		68.5		51.4	
BMI (kg/m^2^)	27.2 ± 3.8	25.1 ± 4.4	26.9 ± 3.3	24.8 ± 4.4	26.5 ± 3.4
Waist circumference (cm)	93.5 ± 12.5	81.0 ± 11.3	96.2 ± 9.6	79.8 ± 11.0	95.2 ± 9.8
Smoking (%)					
Never	31.9	55.0	42.6	50.5	34.4
Former	44.8	25.7	41.6	28.4	41.7
Current	23.3	19.3	15.8	21.1	23.9
Physical activity (%)^a^					
Inactive	12.3	11.6	11.6	9.9	11.8
Moderately inactive	34.3	35.1	36.1	34.5	31.3
Moderately active	27.0	26.5	29.7	29.8	29.6
Active	26.4	26.8	22.6	25.8	27.3
Education level (%)					
Primary school	33.1	24.6	34.2	25.6	25.9
Secondary school	34.4	48.6	32.9	51.2	33.0
University degree	32.5	26.8	32.9	23.2	41.1
NSAID use (%)	9.2	2.5	8.7	2.1	6.0
Age at diagnosis (years)	62.3 ± 7.2	57.5 ± 7.8	64.5 ± 5.2		
Stage at diagnosis (%)					
Local	40.5	62.2	70.7		
Regional	40.5	32.0	24.8		
Distant	19.0	2.2	3.2		
Unknown	-	3.6	1.3		

Values are means ± standard deviations or proportions. ^a^According to the Cambridge physical activity index

**Fig. 1:**
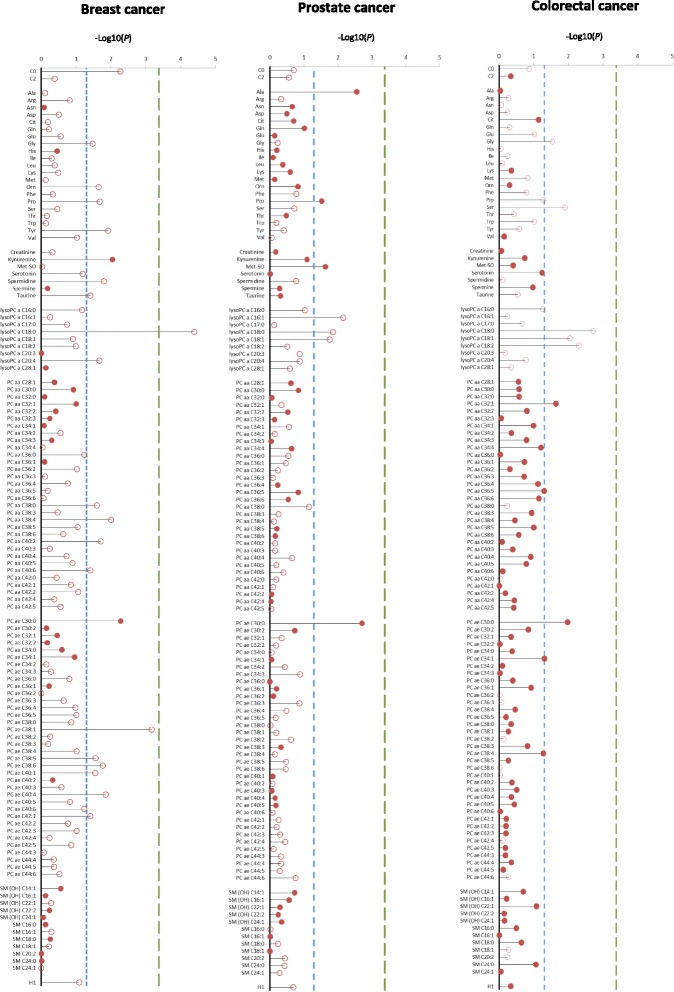
Plasma metabolite concentrations and cancer risk. *P* values from Cox regression analyses on individual metabolite concentrations on the log-2 scale and cancer risk are represented by the needles. The blue dashed lines depict the significance threshold at an uncorrected *P* <0.05 and green dashed lines depict the significance threshold after Bonferroni correction (0.05 divided by 120). Unfilled circles indicate inverse associations and filled circles indicate direct associations. Metabolites are grouped by chemical properties: block 1, acylcarnitines; block 2, amino acids; block 3, biogenic amines; block 4, lysophosphatidylcholines (lysoPCs); block 5, diacylphosphatidylcholines; block 6, acyl-alkyl-phosphatidylcholines; block 7, sphingolipids; and block 8, overall hexoses. All multivariable Cox regression models were adjusted for age, smoking (never, former, current), lifetime alcohol intake (g/d), current aspirin use (yes/no), physical activity (Cambridge index), waist circumference (cm), BMI (continuous), height (cm), and education level (primary school, secondary school, university degree). Analyses on breast cancer risk were additionally adjusted for menopausal status, current HRT use (yes/no), current oral contraceptive use (yes/no), and at least one full term pregnancy (yes/no). Analyses on colorectal cancer risk were additionally adjusted for sex, fiber intake (g/d), and processed meat intake (g/d)

**Fig. 2:**
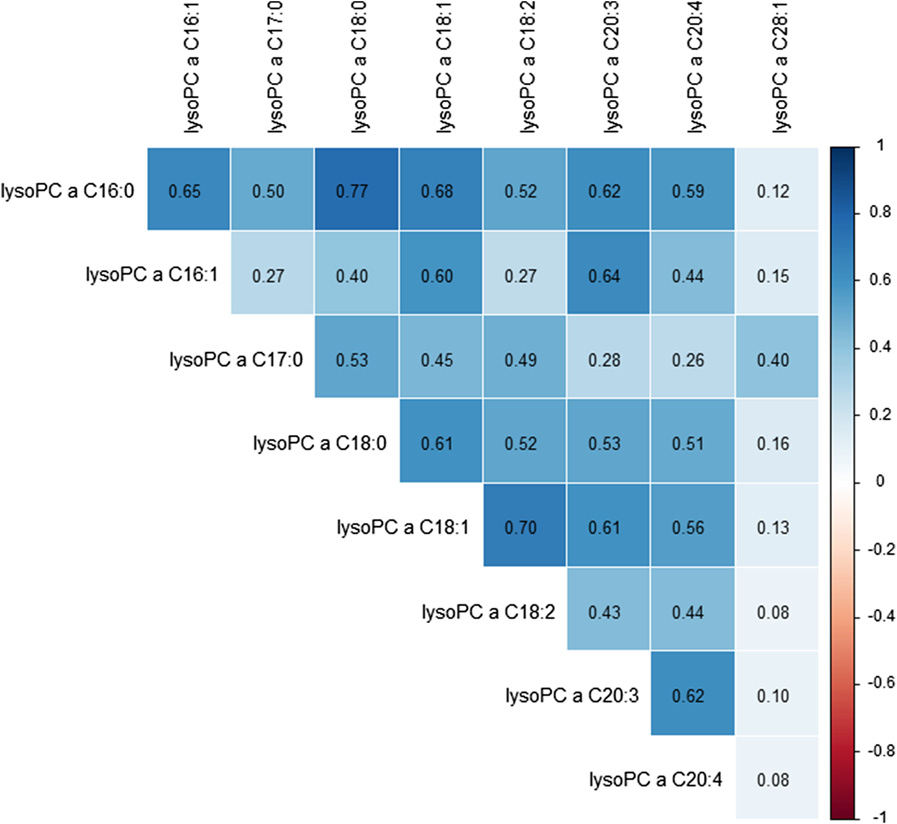
Age- and sex-adjusted Spearman’s correlations between levels of different lysoPCs

Associations between metabolite levels and cancer risk are depicted in Fig. 1. The strongest statistical relationship was found between levels of the lysoPC a C18:0 and breast cancer risk, at a *P* value of 0.00421 corrected for multiple testing by the Bonferroni method. The hazard ratio (HR) of breast cancer among women in the highest quartile of lysoPC a C18:0 concentrations was 0.29 (95 % CI, 0.18–0.47, Table 2). The second and third strongest associations were between PC ae C38:1 and breast cancer risk (HR = 0.53, 95 % CI, 0.34–0.83, *P* = 0.08), and PC ae C30:0 and prostate cancer risk (HR = 1.89, 95 % CI, 1.06–3.36, *P* = 0.23). Considering the consistency of associations between levels of lysoPC a C18:0 and PC ae C30:0 and individual cancer types, visualized in Fig. 1, we decided to expand our analyses to overall cancer risk. LysoPC a C18:0 concentrations were strongly associated with decreased overall cancer risk (HR = 0.37, 95 % CI, 0.27–0.51, *P* <0.0001, Table 2). Similarly, levels of lysoPC a C18:1 and further lysoPCs, which positively correlated with lysoPC a C18:0 levels (Fig. 2), showed inverse associations (Fig. 1, Table 2). Circulating PC ae C30:0 was directly associated with overall cancer risk (HR = 1.85, 95 % CI, 1.31–2.60, *P* = 0.0314, Table 2).

**Table 2:** Hazard ratios (95 % CIs) of cancer across quartiles of lysoPC a C18:0 and PC ae C30:0 concentrations

		Quartile 1	Quartile 2	Quartile 3	Quartile 4	p_trend_ (raw)	p_trend_ (corrected)	Measured in
LysoPC a C18:0								100 %
Breast cancer	Median^a^	13.52	17.34	20.36	23.23			
	n cases	137	96	84	45			
		Ref.	0.63 (0.41, 0.97)	0.65 (0.41, 1.02)	0.29 (0.18, 0.47)	0.00004	0.00421	
Prostate cancer	Median	15.33	18.69	21.43	26.3			
	n cases	93	96	73	48			
		Ref.	1.31 (0.75, 2.28)	0.79 (0.45, 1.39)	0.57 (0.33, 0.98)	0.01388	1	
Colorectal cancer	Median	14.37	17.71	21.21	24.13			
	n cases	47	47	46	23			
		Ref.	1.16 (0.67, 2.01)	1.06 (0.62, 1.82)	0.50 (0.28, 0.90)	0.00196	0.2348	
Overall cancer	Median	14.37	17.93	20.85	24.63			
	n cases	280	228	211	116			
		Ref	0.83 (0.61, 1.12)	0.74 (0.55, 1.00)	0.37 (0.27, 0.51)	1.10 × 10^−9^	1.10 × 10^−7^	
PC ae C30:0								95.6 %
Breast cancer	Median	0.33	0.40	0.47	0.60			
	n cases	69	74	92	113			
		Ref.	1.08 (0.67, 1.75)	1.45 (0.88, 2.37)	1.97 (1.20, 3.23)	0.00522	0.6260	
Prostate cancer	Median	0.28	0.35	0.4	0.53			
	n cases	64	63	71	100			
		Ref.	0.89 (0.50, 1.58)	1.38 (0.78, 2.44)	1.89 (1.06, 3.36)	0.00194	0.2328	
Colorectal cancer	Median	0.28	0.38	0.45	0.59			
	n cases	36	36	46	39			
		Ref	1.00 (0.54, 1.83)	1.79 (1.01, 3.18)	1.84 (1.02, 3.34)	0.01069	1	
Overall cancer	Median	0.30	0.37	0.44	0.58			
	n cases	168	174	208	253			
		Ref	1.03 (0.73, 1.45)	1.41 (1.01, 1.96)	1.85 (1.31, 2.60)	0.00026	0.03137	

Results from Cox proportional hazards regression analyses on pre-diagnostic metabolite concentrations and cancer risk over time. All multivariable Cox models were adjusted for age, smoking (never, former, current), lifetime alcohol intake (g/d), current aspirin use (yes/no), physical activity (Cambridge index), waist circumference (cm), BMI (continuous), height (cm), and education level (primary school, secondary school, university degree). Analyses on breast cancer risk were additionally adjusted for menopausal status, current HRT use (yes/no), current oral contraceptive use (yes/no), and at least one full term pregnancy (yes/no). Analyses on colorectal cancer risk were additionally adjusted for sex, fiber intake (g/d), and processed meat intake (g/d). ^a^Median metabolite concentrations in μmol/L

There was no heterogeneity in the associations between lysoPC a C18:0 or PC ae C30:0 concentrations and cancer risk by lag time (Additional file 2: Table S2). No meaningful associations between metabolite levels and epidemiological covariates such as age, BMI, dietary factors, or smoking status were observed (Tables 3 and 4). Moderate to strong positive correlations were observed between a majority of metabolites within chemical groups (Fig. 2 and Additional file 2: Figure S1).

**Table 3:** Age- and sex-adjusted partial Spearman’s correlations between levels of lysoPC a C18:0 as well as PC ae C30:0 and covariates

	Age	BMI	Height (cm)	Waist (cm)	Fiber intake (g/d)	Meat intake (g/d)
LysoPC a C18:0	−0.01	−0.01	−0.03	−0.02	0.00051	0.06
PC ae C30:0	−0.02	−0.17	0.06	−0.16	0.03	−0.22

**Table 4:** Geometric means (95 % CIs) of metabolite levels adjusted for age and sex across strata of covariates

	LysoPC a C18:0	PC ae C30:0
Smoking status		
Current	19.3 (18.6, 20.1)	0.40 (0.38, 0.41)
Former	19.7 (19.1, 20.3)	0.43 (0.41, 0.44)
Never	19.8 (19.2, 20.3)	0.42 (0.41, 0.44)
Physical activity		
Inactive	19.5 (18.4, 20.7)	0.43 (0.40, 0.45)
Moderately inactive	19.1 (18.4, 19.7)	0.40 (0.39, 0.42)
Moderately active	19.9 (19.2, 20.6)	0.43 (0.42, 0.45)
Active	20.1 (19.4, 20.9)	0.43 (0.41, 0.44)

## Discussion

Since the metabolite levels in our study were measured in blood samples taken years before diagnosis, the present associations between lysoPC and PC ae C30:0 concentrations with the risk of cancer may represent general metabolic alterations fostering the development and growth of cancer cells. In contrast to the relationship between PC ae C30:0 and cancer risk that has not been described in the literature, our findings of associations between lysoPC levels and cancer risk are consistent with results of three case-control studies, in which lower blood concentrations of lysoPCs in patients with breast, prostate and colorectal cancer were found, as compared to controls [16-18].

While a potential shift between lysoPC levels from blood to tumor tissue indicates a higher consumption of lysoPCs by cancer cells, specific signaling properties of lysoPCs in cancer remain to be established [19]. Alternatively, lysoPCs may act as carriers of fatty acids, and extracellular hydrolization of lysoPC a C18:0 and lysoPC a C18:1, followed by a rapid uptake of the respective fatty acids, i.e. stearic (18:0) and oleic acid (18:1), appears to be a characteristic of solid tumors in mice [20]. This is in line with epidemiologic observations of inverse associations between stearic acid levels and breast [21], prostate [3], and colorectal cancer risk [4]. However, it appears spurious why lysoPC a C16:0, lysoPC a C18:1, and lysoPC a C20:4 concentrations also showed inverse associations with cancer risk in our study, whereas circulating palmitic (16:0), oleic (18:1), and arachidonic acid (20:4)—that were not covered by our assay—were not related to cancer risk in previous epidemiological studies.

Possibly, other degradation products of lysoPCs than fatty acids drive tumorigenesis. Extracellular lysoPCs are converted into lysophosphatidic acid (LPA), which induces tumor growth, by autotaxin (ATX), a secreted lysophospholipase D. Overexpression of ATX and LPA receptors has been proposed to be a common feature of several cancers, and both ATX and LPA receptor knockout mice show lower cancer risk [22–24]. Moreover, lysophosphatidylcholine acyltransferase 1 (LPCAT1), which converts lysoPCs into PCs is overexpressed in several cancers, and increased incorporation of PCs into cell membranes may facilitate proliferation, adhesion, and motility of cancer cells [25–27].

Less is known about the role of PC ae C30:0 in carcinogenesis. Elevated PC ae C30:0 concentrations were detected in plasma of patients with ovarian endometriosis [28]. Even though PC levels were inversely associated with prostate cancer risk in one previous study [3] and have been found to be higher in cancer cells than in non-malignant cells [29–31], a distinct biological function of PC ae C30:0 has not been described and ours was the first prospective study on PC ae C30:0 and cancer to our knowledge.

Levels of lysoPCs and PC ae C30:0 were not related to background factors such as BMI, physical activity, or smoking in our study. While PC ae C30:0 has not been shown to be associated with chronic diseases in previous studies, it may seem noteworthy that lysoPC a C18:2 has been proposed to be a potential pre-diagnostic biomarker of diabetes [32]. At the same time, associations between lysoPC a C18:0 and diabetes risk have not been observed in previous studies [32, 33], and potential mediation of associations between lysoPCs and cancer risk by a pre-diabetic state does therefore not appear to be a valid explanation for the present findings.

A limitation of our study is that only a single blood sample was available; however, reasonable mid-term reliability of metabolite levels over time has been demonstrated in reproducibility studies [34–37]. While we cannot provide data on the stability of the analyzed metabolites in long-term storage at −196 °C, it has been shown that freeze-and-thaw cycles do not substantially affect most metabolites covered by the kit we used [38, 39]. Longer exposure to room temperature, which may indeed lead to an increase of lysoPC levels [38], was avoided in our study, and the plasma samples of case patients and non-cases used for the present metabolomics project have been stored and prepared under exactly the same conditions. Many of the metabolites measured in our project are not part of metabolomics platforms previously used in other studies, which hampers comparisons across studies and underlines the need for standardization [1]. Undoubtedly, replication of the present associations is needed before metabolites such as lysoPC a C18:0 or PC ae C30:0 may eventually be used as cancer biomarkers. Moreover, our findings require further investigation in mechanistic studies, before more definite conclusions on lysoPCs and PC ae C30:0 in tumorigenesis can be drawn.

## Conclusion

In summary, we observed consistent associations of lysoPC a C18:0 and PC ae C30:0 concentrations with the risk of three frequent cancer types independent of background factors. Intriguingly, associations in our study did not depend on lag time between blood draw and diagnosis, indicating that low levels of lysoPCs and high levels of PC ae C30:0 are cancer risk factors rather than early markers of disease. The consistency of findings across three cancer types points to global rather than cancer type-specific alterations underlying the observed associations. Further studies are needed to evaluate whether the top hits from our study may have a potential as biomarkers of cancer risk.

## Additional files

Additional file 1:
**Limits of detection, coefficients of variation, and percentages of missing values for individual metabolites.** (XLSX 19 kb) Additional file 2:
**Supplementary main results on hazard ratios of cancer across quartiles of metabolite levels (Table S1 as an extension of Fig.** **1** **), results of evaluations of associations between metabolites and cancer risk by lag time (Table S2), and correlations between metabolite concentrations by chemical group (Figure S1).** (PDF 586 kb)

## Abbreviations

ATX: autotaxin
BMI: body mass index
CI: confidence interval
EPIC: European Prospective Investigation into Cancer and Nutrition
FIA-MS/MS: flow injection analysis-tandem mass spectrometry
HR: hazard ratio
HRT: hormone replacement therapy
ICD: International Classification of Diseases
LC-MS/MS: liquid chromatography-tandem mass spectrometry
LPA: lysophosphatidic acid
lysoPC: lysophosphatidylcholine
NSAID: nonsteroidal anti-inflammatory drug
PC: phosphatidylcholine
PITC: phenylisothiocyanate
QC: quality control
