# Supplementary material for: Higher plasma levels of lysophosphatidylcholine 18:0 are related to a lower risk of common cancers in a prospective metabolomics study
Source: BMC Med. 2016 Jan 28;14:13. doi: 10.1186/s12916-016-0552-3 (PMC4730724; doi:10.1186/s12916-016-0552-3)
Supplement: Additional file 2: — Supplementary main results on hazard ratios of cancer across quartiles of metabolite levels (Table S1 as an extension of Fig. 1 ), results of evaluations of associations between metabolites and cancer risk by lag time (Table S2), and correlations between metabolite concentrations by chemical group (Figure S1). (PDF 586 kb) [file 12916_2016_552_MOESM2_ESM.pdf]

Additional File 2

Supplementary Figures

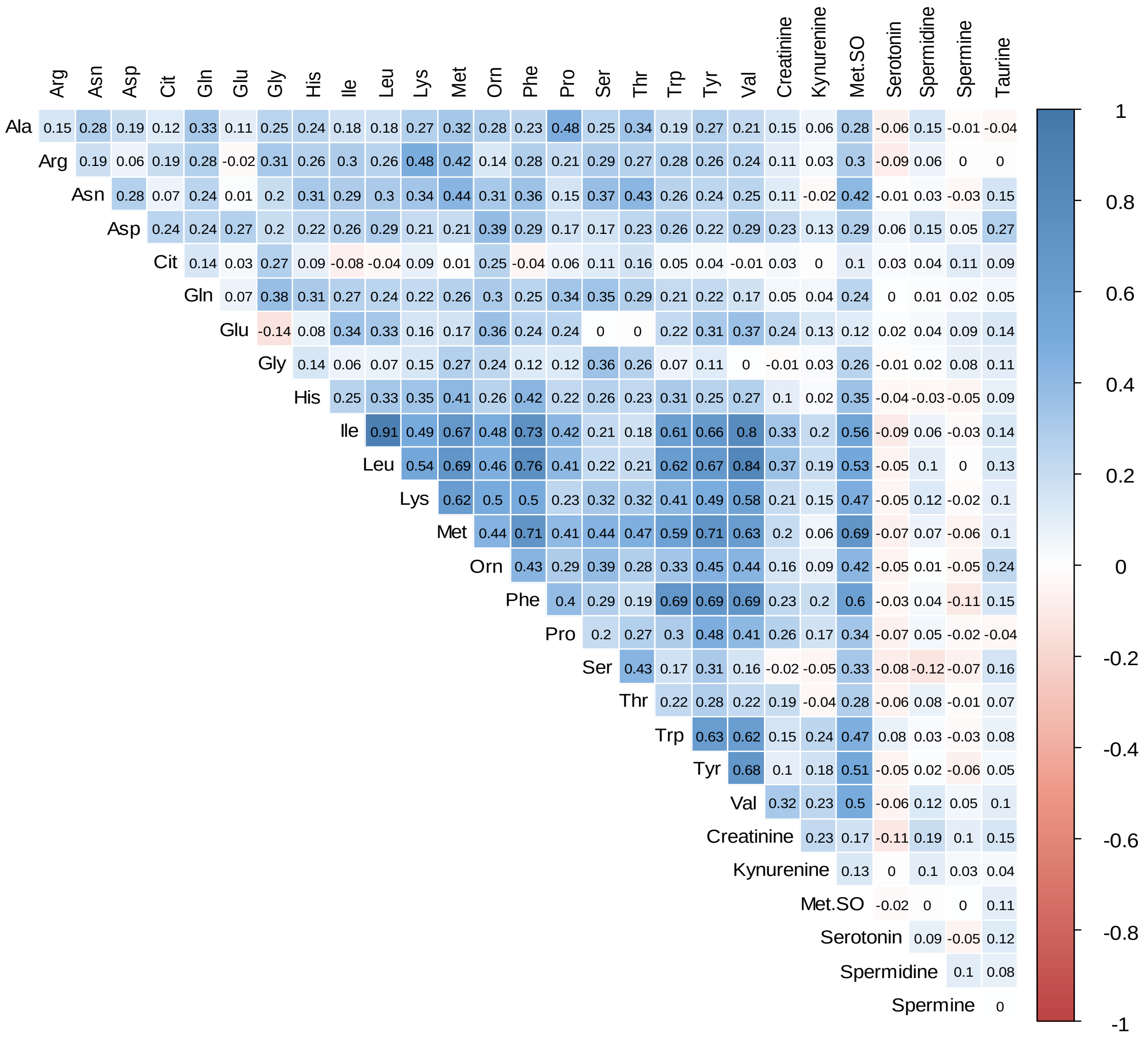

Figure S1a Age- and sex-adjusted Spearman’s correlations between plasma metabolite levels (amino acids and biogenic amines), related to Figure 2.

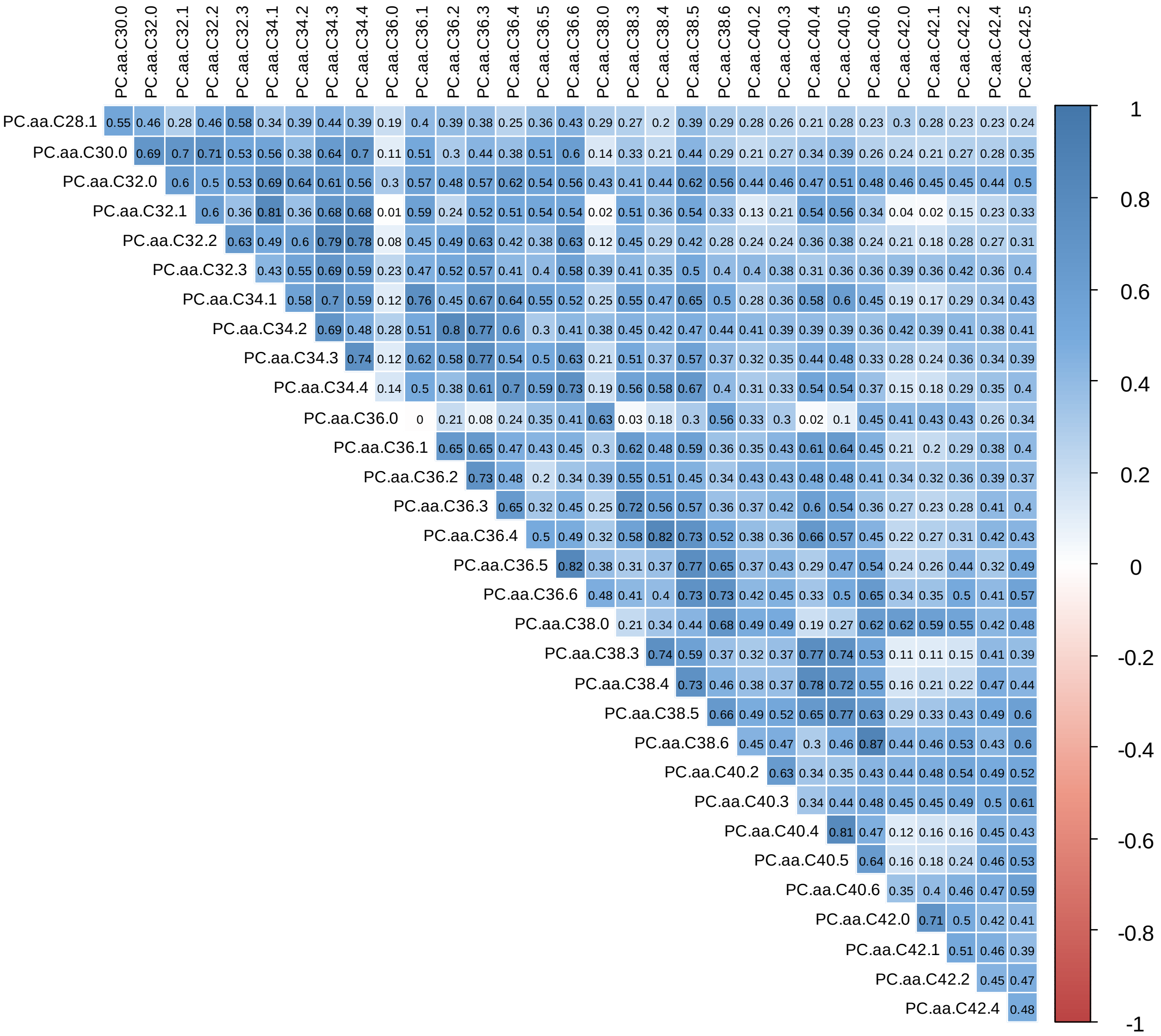

Figure S1b Age- and sex-adjusted Spearman's correlations between plasma metabolite levels (diacyl-phosphatidylcholines), related to Figure 2.

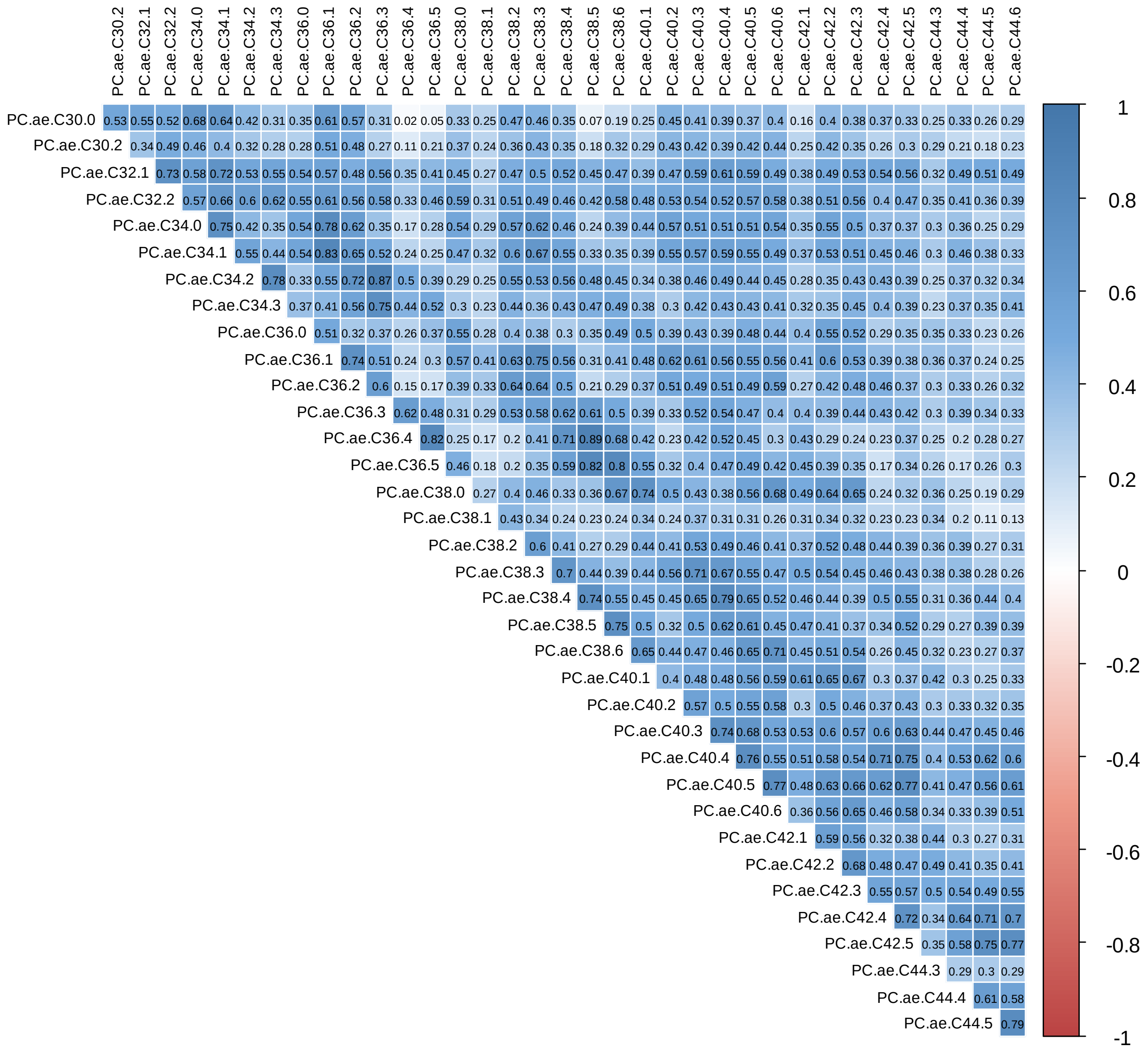

Figure S1c Age- and sex-adjusted Spearman's correlations between plasma metabolite levels (acyl-alkyl-phosphatidylcholines), related to Figure 2.

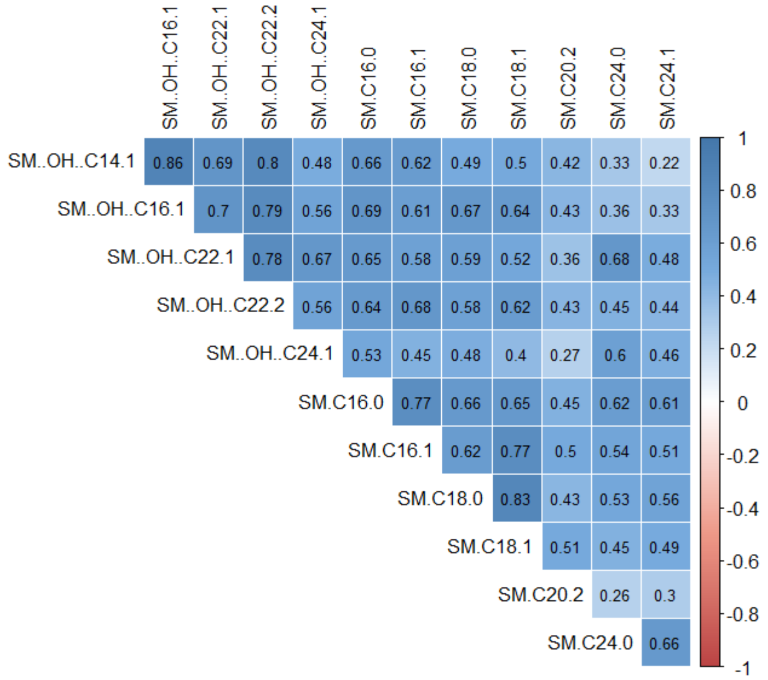

Figure S1d Age- and sex-adjusted Spearman's correlations between plasma metabolite levels (sphingolipids), related to Figure 2.

Supplementary Tables

Table S1a Hazard ratios for overall cancer across quartiles of metabolite levels (10 strongest associations), related to Figure 1.

		Quartile1	Quartile 2	Quartile 3	Quartile 4	P trend (raw)	P trend (corrected)	Measured in
LysoPC a C18:0	n cases	280	228	211	116			100 %
	median*	14.37	17.93	20.85	24.63			
		Ref	0.83 (0.61,1.12)	0.74 (0.55,1.00)	0.37 (0.27,0.51)	$1.10*10^{-9}$	$1.10*10^{-7}$	
LysoPC a C18:1	n cases	275	227	174	159			100 %
	median	10.48	13.01	15.43	19.15			
		Ref	0.87 (0.64,1.18)	0.62 (0.45,0.84)	0.58 (0.42,0.80)	0.00007	0.00792	
PC ae C30:0	n cases	168	174	208	253			95.6 %
	median	0.3	0.37	0.44	0.58			
		Ref	1.03 (0.73.1.45)	1.41 (1.01.1.96)	1.85 (1.31.2.60)	0.00026	0.03137	
LysoPC a C20:4	n cases	254	212	227	142			100 %
	median	3.89	5.16	6.12	7.88			
		Ref	0.72 (0.53,0.97)	0.86 (0.63,1.17)	0.49 (0.34,0.69)	0.00042	0.05038	
LysoPC a C16:0	n cases	253	206	218	158			100 %
	median	48.74	58.11	65.69	77.38			
		Ref	0.75 (0.56,1.02)	0.95 (0.70,1.29)	0.51 (0.37,0.71)	0.00054	0.06532	
C0	n cases	242	196	176	204			97.7 %
	median	24.01	28.92	33.12	39.16			
		Ref	0.70 (0.51,0.96)	0.60 (0.43,0.83)	0.58 (0.42,0.81)	0.00073	0.0879	
Glycine	n cases	273	198	195	169			100 %
	median	133	172	204	260			
		Ref	0.67 (0.49,0.90)	0.71 (0.52,0.96)	0.56 (0.41,0.78)	0.00137	0.1642	
PC aa C38:0	n cases	223	215	204	193			100 %
	median	1.69	2.11	2.48	3.08			
		Ref	1.02 (0.75,1.39)	0.89 (0.65,1.21)	0.71 (0.52,0.98)	0.00164	0.1964	
Tyrosine	n cases	216	218	172	203			96.9 %
	median	42.95	54.8	65.3	80.7			
		Ref	0.90 (0.65,1.24)	0.65 (0.47,0.90)	0.71 (0.51,0.99)	0.00182	0.2181	
LysoPC a C18:2	n cases	283	216	167	169			100 %
	median	17.16	23.07	28.71	35.95			
		Ref	0.79 (0.58,1.07)	0.67 (0.48,0.93)	0.61 (0.43,0.86)	0.00331	0.3972	

Results from Cox proportional hazards regression analyses on pre-diagnostic metabolite concentrations and cancer risk over time. All multivariable Cox regression models were adjusted for age, sex, smoking (never, former, current), lifetime alcohol intake (g/d), current aspirin use (yes/no), physical activity (Cambridge Index), waist circumference (cm), BMI (continuous), height (cm), education level (primary school, secondary school, university degree), fiber intake (g/d), processed meat intake (g/d);
*Median metabolite concentrations in µmol/L;

Table S1b Hazard ratios for breast cancer across quartiles of metabolite levels (10 strongest associations), related to Figure 1.

		Quartile1	Quartile 2	Quartile 3	Quartile 4	P trend (raw)	P trend (corrected)	Measured in
LysoPC a C18:0	n cases	137	96	84	45			100 %
	median*	13.52	17.34	20.36	23.23			
		Ref.	0.63 (0.41,0.97)	0.65 (0.41,1.02)	0.29 (0.18,0.47)	0.00004	0.00421	
PC ae C38:1	n cases	130	56	93	77			98.1 %
	median	0.56	0.84	1.02	1.36			
		Ref.	0.49 (0.31,0.77)	0.73 (0.47,1.13)	0.53 (0.34,0.83)	0.00067	0.08036	
PC ae C30:0	n cases	69	74	92	113			95.6 %
	median	0.33	0.4	0.47	0.6			
		Ref.	1.08 (0.67,1.75)	1.45 (0.88,2.37)	1.97 (1.20,3.23)	0.00522	0.62608	
C0	n cases	100	107	71	71			97.7 %
	median	21.79	26.48	30.46	36.66			
		Ref.	0.96 (0.62,1.50)	0.62 (0.38,1.01)	0.54 (0.33,0.89)	0.00538	0.6458	
Kynurenine	n cases	49	65	63	104			77.6 %
	median	1.98	2.5	2.58	3.52			
		Ref.	1.27 (0.74,2.18)	1.13 (0.64,2.01)	1.97 (1.17,3.33)	0.00889	1	
PC aa C38:4	n cases	109	94	91	68			100 %
	median	64.73	82.08	98.84	118.16			
		Ref.	0.92 (0.60,1.41)	0.81 (0.52,1.26)	0.54 (0.33,0.88)	0.00991	1	
Tyrosine	n cases	112	88	79	72			96.9 %
	median	40.8	53.05	62.4	82.2			
		Ref.	0.76 (0.48,1.22)	0.54 (0.34,0.88)	0.57 (0.35,0.93)	0.0119	1	
PC ae C40:4	n cases	123	87	87	65			100 %
	median	1.59	1.87	2.14	2.62			
		Ref.	0.70 (0.45,1.08)	0.60 (0.38,0.94)	0.47 (0.29,0.76)	0.01415	1	
Spermidine	n cases	94	76	74	62			85.3 %
	median	1.08	1.04	1.08	1.15			
		Ref.	0.79 (0.49,1.28)	0.71 (0.43,1.16)	0.61 (0.38,0.98)	0.01592	1	
PC ae C38:6	n cases	107	93	91	71			100 %
	median	4.55	5.55	6.16	7.89			
		Ref.	0.75 (0.48,1.19)	0.87 (0.54,1.38)	0.56 (0.35,0.89)	0.01692	1	

Results from Cox proportional hazards regression analyses on pre-diagnostic metabolite concentrations and cancer risk over time. Cox regression models were adjusted for age, smoking (never, former, current), lifetime alcohol intake (g/d), current aspirin use (yes/no), physical activity (Cambridge Index), waist circumference (cm), BMI (continuous), height (cm), education level (primary school, secondary school, university degree), menopausal status, current HRT use (yes/no), current oral contraceptive use (yes/no), at least one full term pregnancy (yes/no);
*Median metabolite concentrations in µmol/L;

Table S1c Hazard ratios for prostate cancer across quartiles of metabolite levels (10 strongest associations), related to Figure 1.

		Quartile1	Quartile 2	Quartile 3	Quartile 4	P trend (raw)	P trend (corrected)	Measured in
PC ae C30:0	n cases	64	63	71	100			95.6 %
	median*	0.28	0.35	0.4	0.53			
		Ref.	0.89 (0.50,1.58)	1.38 (0.78,2.44)	1.89 (1.06,3.36)	0.00194	0.2328	
Alanine	n cases	57	62	94	97			99.9 %
	median	251	313	364.5	450			
		Ref.	0.96 (0.54,1.71)	1.45 (0.84,2.50)	1.66 (0.98,2.81)	0.00276	0.3318	
LysoPC a C16:1	n cases	95	81	68	66			100 %
	median	1.43	1.79	2.19	2.78			
		Ref.	0.93 (0.55,1.59)	0.62 (0.36,1.04)	0.70 (0.41,1.18)	0.00706	0.8471	
LysoPC a C18:0	n cases	93	96	73	48			100 %
	median	15.33	18.69	21.43	26.3			
		Ref.	1.31 (0.75,2.28)	0.79 (0.45,1.39)	0.57 (0.33,0.98)	0.01388	1	
LysoPC a C18:1	n cases	116	79	62	53			100 %
	median	11.65	13.88	15.95	19.86			
		Ref.	0.83 (0.50,1.37)	0.64 (0.38,1.07)	0.48 (0.28,0.82)	0.1717	1	
Met-SO	n cases	43	77	88	94			97.0 %
	median	1.6	1.71	1.84	2.35			
		Ref.	1.42 (0.79,2.55)	2.44 (1.32,4.48)	1.71 (0.95,3.08)	0.02291	1	
Proline	n cases	62	88	78	82			100 %
	median	128,5	171	207,5	260			
		Ref.	1.43 (0.82,2.51)	1.29 (0.74,2.25)	1.94 (1.11,3.40)	0.02955	1	
PC aa C38:0	n cases	85	80	70	75			100 %
	median	1.65	2.03	2.38	2.93			
		Ref.	1.02 (0.61,1.70)	0.64 (0.38,1.08)	0.74 (0.43,1.26)	0.07045	1	
Kynurenine	n cases	59	51	59	80			77.6 %
	median	2.18	2.43	2.84	3.59			
		Ref.	1.31 (0.68,2.53)	1.24 (0.66,2.36)	1.63 (0.88,3.02)	0.08071	1	
LysoPC a C16:0	n cases	88	85	92	45			100 %
	median	53.14	60.34	69.86	84.45			
		Ref.	1.01 (0.61,1.69)	1.31 (0.76,2.24)	0.46 (0.26,0.81)	0.09339	1	

Results from Cox proportional hazards regression analyses on pre-diagnostic metabolite concentrations and cancer risk over time. Cox regression models were adjusted for age, smoking (never, former, current), lifetime alcohol intake (g/d), current aspirin use (yes/no), physical activity (Cambridge Index), waist circumference (cm), BMI (continuous), height (cm), education level (primary school, secondary school, university degree);
*Median metabolite concentrations in µmol/L;

Table S1d Hazard ratios for colorectal cancer across quartiles of metabolite levels (10 strongest associations), related to Figure 1.

		Quartile1	Quartile 2	Quartile 3	Quartile 4	P trend (raw)	P trend (corrected)	Measured in
LysoPC a C18:0	n cases	47	47	46	23			100 %
	median*	14.37	17.71	21.21	24.13			
		Ref	1.16 (0.67,2.01)	1.06 (0.62,1.82)	0.50 (0.28,0.90)	0.00196	0.2348	
LysoPC a C18:2	n cases	67	38	34	24			100 %
	median	16.64	22.78	28.16	36.32			
		Ref	0.61 (0.37,1.02)	0.64 (0.36,1.13)	0.42 (0.23,0.78)	0.00503	0.6031	
LysoPC a C18:1	n cases	54	43	36	30			100 %
	median	10.43	13.11	15.29	19.26			
		Ref	0.99 (0.59,1.67)	0.78 (0.46,1.33)	0.58 (0.33,1.02)	0.0092	1	
PC ae C30:0	n cases	36	36	46	39			95.6 %
	median	0.28	0.38	0.45	0.59			
		Ref	1.00 (0.54,1.83)	1.79 (1.01,3.18)	1.84 (1.02,3.34)	0.01069	1	
Serine	n cases	56	43	45	19			100 %
	median	62.25	85.1	102	120			
		Ref	0.77 (0.48,1.26)	0.78 (0.48,1.28)	0.42 (0.22,0.78)	0.01264	1	
PC aa C32:1	n cases	25	29	48	61			100 %
	median	10.02	14.33	18.49	34.43			
		Ref	1.03 (0.54,1.97)	1.76 (0.98,3.17)	1.71 (0.95,3.08)	0.02308	1	
Glycine	n cases	61	39	38	25			100 %
	median	134	169	209	253			
		Ref	0.61 (0.37,1.00)	0.68 (0.41,1.12)	0.46 (0.26,0.82)	0.02929	1	
PC ae C34:1	n cases	35	39	44	45			100 %
	median	6.39	8.07	9.05	11.25			
		Ref	1.38 (0.79,2.40)	1.80 (1.02,3.17)	1.65 (0.92,2.97)	0.04893	1	
PC aa C36:5	n cases	28	35	49	51			100 %
	median	14.27	18.68	25.69	40.74			
		Ref	1.26 (0.69,2.29)	1.51 (0.86,2.66)	1.53 (0.87,2.72)	0.05039	1	
PC ae C38:4	n cases	31	55	36	41			100 %
	median	8.62	10.13	11.62	14.21			
		Ref	1.80 (1.07,3.01)	1.45 (0.83,2.53)	1.34 (0.78,2.30)	0.05299	1	

Results from Cox proportional hazards regression analyses on pre-diagnostic metabolite concentrations and cancer risk over time. All multivariable Cox regression models were adjusted for age, sex, smoking (never, former, current), lifetime alcohol intake (g/d), current aspirin use (yes/no), physical activity (Cambridge Index), waist circumference (cm), BMI (continuous), height (cm), education level (primary school, secondary school, university degree), fiber intake (g/d), processed meat intake (g/d);
*Median metabolite concentrations in µmol/L;

Table S2a Hazard ratios of cancer across quartiles of LysoPC a C18:0 levels stratified by median lag time, related to Table 2.

		Quartile 1	Quartile 2	Quartile 3	Quartile 4	P trend (raw)	P trend (corr.)	P hetero-geneity
**Overall Cancer**								0.5534
Lag time <6.48y	n cases	150	113	97	57			
		Ref	0.76 (0.53,1.09)	0.69 (0.48,0.98)	0.36 (0.24,0.54)	$4.65*10^{-8}$	$5.58*10^{-6}$	
Lag time ≥6.48y	n cases	130	115	114	59			
		Ref	1.05 (0.71,1.57)	0.87 (0.58,1.28)	0.40 (0.26,0.62)	$1.06*^{10-5}$	0.00127	
**Breast Cancer**								0.6460
Lag time <6.36y	n cases	77	41	38	26			
		Ref	0.44 (0.25,0.79)	0.52 (0.29,0.91)	0.29 (0.16,0.52)	0.00015	0.0176	
Lag time ≥6.36y	n cases	60	55	46	19			
		Ref	0.77 (0.45,1.31)	0.75 (0.42,1.33)	0.24 (0.12,0.47)	0.00097	0.1161	
**Prostate Ca.**								0.4921
Lag time <6.83y	n cases	44	52	39	20			
		Ref	1.41 (0.72,2.77)	1.05 (0.52,2.10)	0.57 (0.28,1.16)	0.07301	1	
Lag time ≥6.83y	n cases	49	44	34	28			
		Ref	1.11 (0.55,2.21)	0.47 (0.23,0.93)	0.49 (0.24,1.00)	0.00953	1	
**Colorectal Ca.**								
Lag time <6.57y	n cases	27	25	17	12			0.0119
		Ref	0.94 (0.50,1.77)	0.65 (0.33,1.29)	0.39 (0.18,0.82)	0.00037	0.04427	
Lag time ≥6.57y	n cases	20	22	29	11			
		Ref	1.81 (0.83,3.95)	2.51 (1.29,4.85)	0.88 (0.38,1.99)	0.7794	1	

Results from Cox proportional hazards regression analyses on pre-diagnostic metabolite concentrations and cancer risk over time.
Cox regression analyses were adjusted for the following factors:
Overall cancer: Age, sex, height (cm), waist (cm), smoking (never, former, current), lifetime alcohol intake (g/d), current aspirin use (yes/no), physical activity (Cambridge Index), education level (primary school, secondary school, university degree), fiber intake (g/d), processed meat intake (g/d);
Breast cancer: Age, height (cm), waist (cm), smoking (never, former, current), lifetime alcohol intake (g/d), physical activity (Cambridge Index), education level (primary school, secondary school, university degree), menopausal status, current HRT use (yes/no), current oral contraceptive use (yes/no), at least one full term pregnancy (yes/no);
Colon cancer: Age, sex, waist (cm), smoking (never, former, current), lifetime alcohol intake (g/d);
Prostate cancer: Age, height (cm), waist (cm), physical activity (Cambridge Index), smoking (never, former, current), lifetime alcohol intake (g/d), education level (primary school, secondary school, university degree);

Table S2b Hazard ratios for cancer across quartiles of PC ae C30:0 levels stratified by median lag time, related to Table 2.

		Quartile 1	Quartile 2	Quartile 3	Quartile 4	P trend (raw)	P trend (corr.)	P hetero-geneity
**Overall Cancer**								0.7256
Lag time <6.48y	n cases	90	71	108	129			
		Ref	0.75 (0.49,1.15)	1.37 (0.92,2.06)	1.70 (1.13,2.55)	0.00106	0.1269	
Lag time ≥6.48y	n cases	78	103	100	124			
		Ref	1.71 (1.10,2.66)	1.91 (1.22,3.00)	2.62 (1.64,4.19)	0.00051	0.0607	
**Breast Cancer**								0.8907
Lag time <6.36y	n cases	33	33	49	59			
		Ref	0.95 (0.51,1.76)	1.41 (0.74,2.71)	2.01 (1.07,3.76)	0.01414	1	
Lag time ≥6.36y	n cases	36	41	43	54			
		Ref	1.30 (0.69,2.44)	1.52 (0.80,2.90)	1.99 (1.01,3.91)	0.03792	1	
**Prostate Ca.**								0.7736
Lag time <6.83y	n cases	36	25	38	50			
		Ref	0.55 (0.26,1.15)	1.39 (0.68,2.85)	1.85 (0.91,3.73)	0.0045	0.5399	
Lag time ≥6.83y	n cases	28	38	33	50			
		Ref	1.35 (0.68,2.70)	1.35 (0.64,2.87)	1.91 (0.92,3.98)	0.01628	1	
**Colorectal Ca.**								0.3988
Lag time <6.57y	n cases	19	16	24	17			
		Ref	0.89 (0.42,1.90)	1.68 (0.86,3.29)	1.34 (0.65,2.75)	0.1649	1	
Lag time ≥6.57y	n cases	17	20	22	22			
		Ref	1.65 (0.75,3.62)	2.60 (1.13,5.95)	2.54 (1.09,5.92)	0.01935	1	

Results from Cox proportional hazards regression analyses on pre-diagnostic metabolite concentrations and cancer risk over time. Cox regression analyses were adjusted for the following factors:
Overall cancer: Age, sex, height (cm), waist (cm), smoking (never, former, current), lifetime alcohol intake (g/d), current aspirin use (yes/no), physical activity (Cambridge Index), education level (primary school, secondary school, university degree), fiber intake (g/d), processed meat intake (g/d);
Breast cancer: Age, height (cm), waist (cm), smoking (never, former, current), lifetime alcohol intake (g/d), physical activity (Cambridge Index), education level (primary school, secondary school, university degree), menopausal status, current HRT use (yes/no), current oral contraceptive use (yes/no), at least one full term pregnancy (yes/no);
Colon cancer: Age, sex, waist (cm), smoking (never, former, current), lifetime alcohol intake (g/d);
Prostate cancer: Age, height (cm), waist (cm), physical activity (Cambridge Index), smoking (never, former, current), lifetime alcohol intake (g/d), education level (primary school, secondary school, university degree);

Table S2c Hazard ratios for cancer across quartiles of metabolite levels after exclusion of cases from the first two years of follow-up, related to Table 2.

		Quartile 1	Quartile 2	Quartile 3	Quartile 4	P trend (raw)	P trend (corr.)
**Overall cancer**							
LysoPC a C18:0	n cases	242	199	188	100		
		Ref	0.85 (0.62,1.16)	0.75 (0.55,1.02)	0.36 (0.25,0.50)	$1.01*10^{-9}$	$1.21*10^{-7}$
PC ae C30:0	n cases	147	156	181	218		
		Ref	1.11 (0.78,1.57)	1.45 (1.03,2.05)	1.91 (1.34,2.73)	0.00051	0.06140
**Breast cancer**							
LysoPC a C18:0	n cases	115	88	71	34		
		Ref	0.68 (0.44,1.06)	0.64 (0.40,1.02)	0.25 (0.15,0.42)	0.00002	0.00187
PC ae C30:0	n cases	60	65	79	92		
		Ref	1.11 (0.67,1.83)	1.46 (0.88,2.42)	1.86 (1.11,3.10)	0.02463	1
**Prostate cancer**							
LysoPC a C18:0	n cases	81	86	68	45		
		Ref	1.42 (0.81,2.47)	0.81 (0.46,1.43)	0.59 (0.34,1.04)	0.01265	1
PC ae C30:0	n cases	55	59	65	90		
		Ref	0.99 (0.55,1.79)	1.51 (0.85,2.68)	2.01 (1.12,3.60)	0.00139	0.1669
**Colon cancer**							
LysoPC a C18:0	n cases	39	41	41	20		
		Ref	1.30 (0.71,2.35)	1.20 (0.67,2.15)	0.55 (0.29,1.04)	0.01685	1
PC ae C30:0	n cases	30	34	36	37		
		Ref	1.20 (0.64,2.28)	1.71 (0.90,3.26)	2.21 (1.17,4.17)	0.00494	0.5925

Results from Cox proportional hazards regression analyses on pre-diagnostic metabolite concentrations and cancer risk over time. Cox regression analyses were adjusted for the following factors:
Overall cancer: Age, sex, height (cm), waist (cm), smoking (never, former, current), lifetime alcohol intake (g/d), current aspirin use (yes/no), physical activity (Cambridge Index), education level (primary school, secondary school, university degree), fiber intake (g/d), processed meat intake (g/d);
Breast cancer: Age, height (cm), waist (cm), smoking (never, former, current), lifetime alcohol intake (g/d), physical activity (Cambridge Index), education level (primary school, secondary school, university degree), menopausal status, current HRT use (yes/no), current oral contraceptive use (yes/no), at least one full term pregnancy (yes/no);
Colon cancer: Age, sex, waist (cm), smoking (never, former, current), lifetime alcohol intake (g/d);
Prostate cancer: Age, height (cm), waist (cm), physical activity (Cambridge Index), smoking (never, former, current), lifetime alcohol intake (g/d), education level (primary school, secondary school, university degree);
